# A Succession of MRI Scans Supports the Diagnosis of Lumbar Ligamentum Flavum Hematoma: A Case Report and Review of the Literature

**DOI:** 10.1155/2018/2860621

**Published:** 2018-11-27

**Authors:** Yuyu Ishimoto, Mamoru Kawakami, Elizabeth Curtis, Cyrus Cooper, Nami Moriguchi, Yukihiro Nakagawa

**Affiliations:** ^1^Spine Care Center, Wakayama Medical University Kihoku Hospital, Katsuragi-cho 649-7113, Japan; ^2^MRC Lifecourse Epidemiology Unit, Southampton General Hospital, Southampton SO166YD, UK; ^3^Arthritis Research UK/MRC Centre for Musculoskeletal Work and Health, Southampton General Hospital, Southampton SO166YD, UK; ^4^National Institute for Health Research (NIHR) Oxford Biomedical Research Centre, University of Oxford, UK

## Abstract

Ligamentum flavum hematoma (LFH) is a rare cause of spinal nerve compression. This condition remains challenging to diagnose using MRI due to the changing intensity of the hematoma on imaging. The aim of this study was to describe the patient with LFH who had a succession of MRI scans carried out. We report on a 71-year-old woman with a mass at L4/5 and decompression surgery was performed for her left leg symptom. She had MRI carried out in a previous hospital and also had MRI again in our hospital. In a 2^nd^ MRI of the same area, after a 2-week interval, a newly isointense mass was present within the anterior part of the previously identified lesion on T1-weighted image and the hyperintense area in the lesion was a little extended on T2-weighted imaging. Her symptoms were resolved immediately after decompression surgery. Following a review of previous cases, we suggest that consecutive MRI scanning may support the diagnostic process for LFH.

## 1. Introduction

Various pathological changes of the ligamentum flavum have been identified, including hypertrophy, calcification, ossification, and cyst formation that may compress the spinal cord. Ligamentum flavum hematoma (LFH) is a rare cause of spinal nerve compression [1, 2]. Around 30 cases of lumbar LFH have been previously described in the literatures. However, its pathogenesis remains unclear [3–5] and this condition remains challenging to diagnose using MRI due to the changing intensity of the hematoma on imaging [5, 6]. Here, we describe a 71-year-old woman with LFH whose symptoms resolved after L4/5 decompression surgery. This is the first case to describe comparative MRI scans over time, as MRI was carried out in the previous hospital and repeated in our hospital. Clinically relevant changes were identified between the consecutive MRIs.

## 2. Case Presentation

A 71-year-old woman was referred for evaluation of back pain and worsening left lower extremity pain, which included neurogenic claudication. She gave a history of a fall from standing height, with onset of low back pain. Around a month later, her left leg pain became a prominent feature, with a symptom duration of approximately five months at the time of injury. She did not experience lumbar epidural or intrathecal injections. She reported no history of fever and no difficulty passing urine. Significant medical history included mild hypertension, of note, and there was no history of malignancy and no history of use of anticoagulant drugs. Both platelet count and prothrombin time were in the normal range. On examination, there was full power and symmetrical reflexes in both lower extremities. Patchy reduction in sensation to the left lower limb was noted. She was given a left L5 nerve root block which was only effective for 3 days. The patient and/or her families were informed that data from the case would be submitted for publication and gave their consent.

### 2.1. MRI

**Figure 1:**
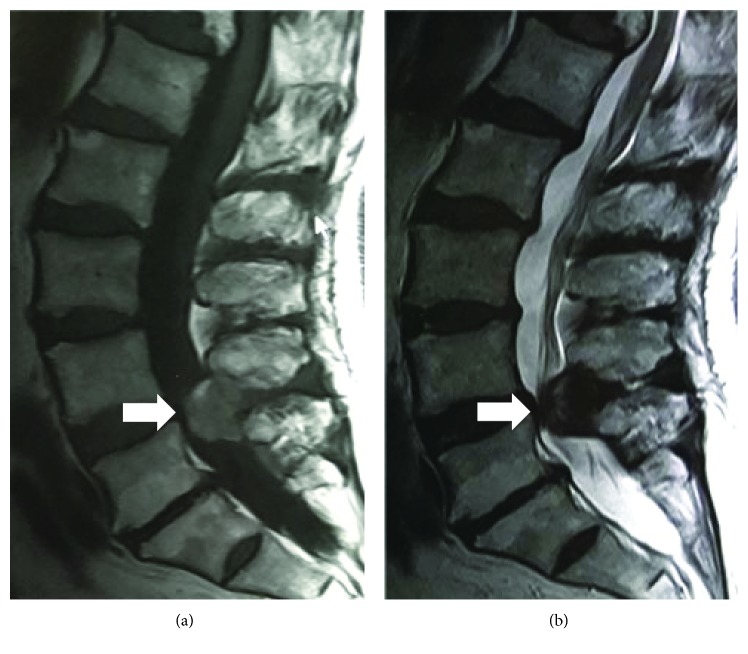
Magnetic resonance images showing a posterior epidural mass at L4/5 compressing the thecal sac and spinal cord and linking with the ligamentum flavum.

**Figure 2:**
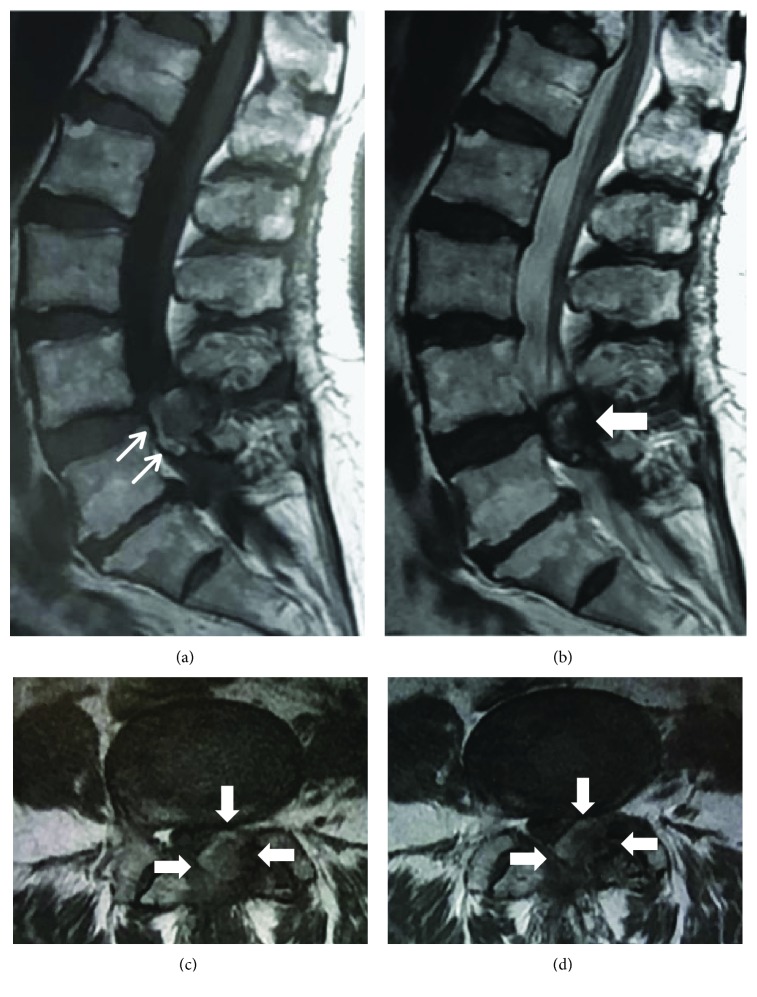
2^nd^ magnetic resonance images (MRI) showing left posterior mass at the same area of the 1^st^ MRI. (a) Sagittal T1-weighted axial MRI reveals that there was a newly isointense mass present within the anterior part of the previously identified lesion. (b) Sagittal T2-weighted axial MRI reveals that the intensity inside the mass was a little extended. (c, d) T1-weighted axial MRI at L4/5 reveals the well-defined extradural mass in the left posterior aspect of the thecal sac. (c) There was no significant enhancement with Gd-based contrast (d).

A lumbar MRI without gadolinium (Gd) had been performed prior to evaluation (4 months after symptom onset) in a previous hospital (Figure 1). The MRI showed a posterior mass at L4/5. On T1-weighted images (Figure 1(a)), the mass was isointense, with a few hyperintense areas within. On T2-weighted images, the mass was hyperintense in the center and hypointense in the periphery (Figure 1(b)). We suspected the spinal tumour and carried out a 2^nd^ MRI with Gd. On the 2^nd^ MRI of the same area (Figures 2(a)–2(c)), after a 2-week interval, a newly isointense mass was present within the anterior part of the previously identified lesion on T1-weighted image (Figure 1(a)) and the hyperintense area in the lesion was a little extended on T2-weighted imaging (Figure 2(b)). There was no significant enhancement with Gd-based contrast (Figures 2(c) and 2(d)). Her symptoms were not changed between the 1^st^ and 2^nd^ MRI.

### 2.2. Surgery

**Figure 3:**
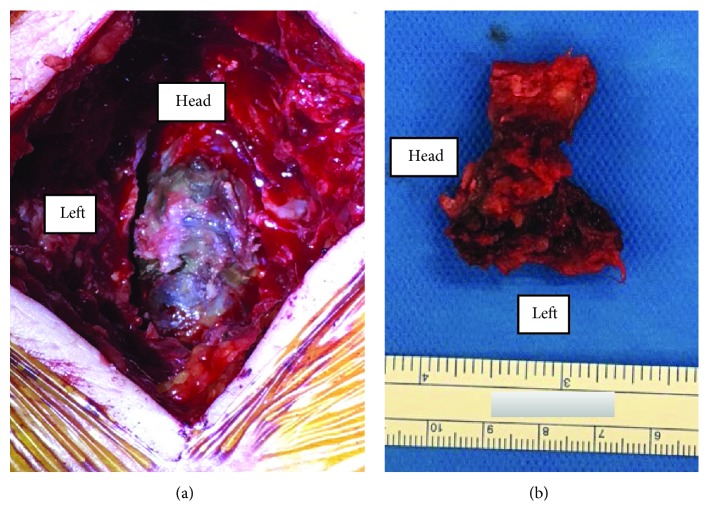
(a) The ligamentum flavum after L4/5 partial laminectomy. (b) The solid blackish, swelling ligament in left side.

**Figure 4:**
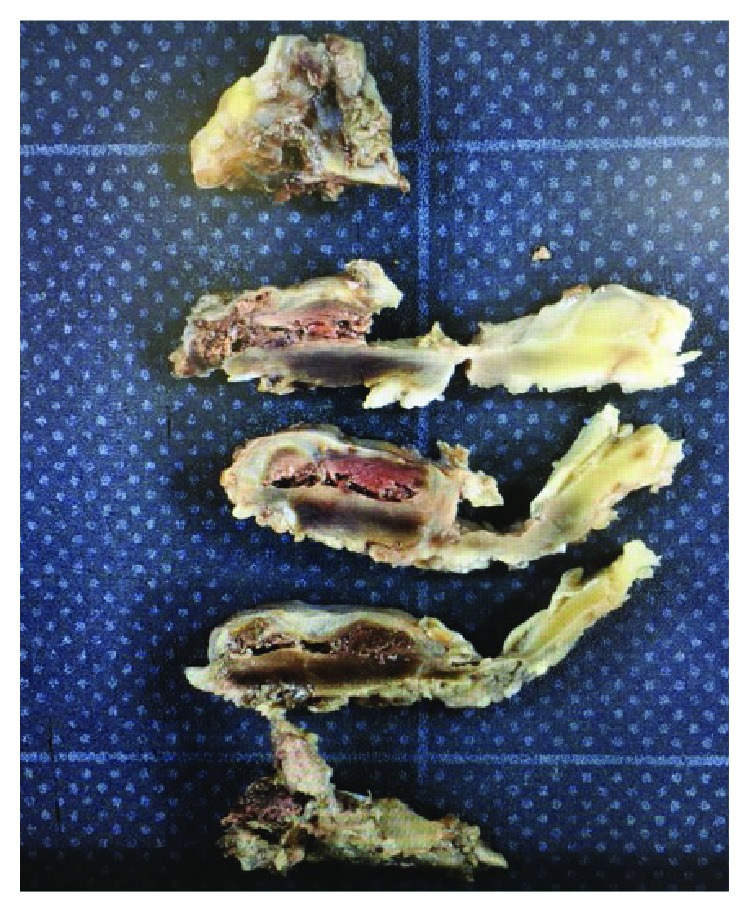
A hematoma inside the removal ligament.

The patient underwent surgery for decompression of the spinal canal and resection of the lesion, which at this stage was presumed to be an epidural tumour. After L4/5 partial laminectomy, the solid blackish ligamentum flavum was visible and firmly adherent to the dural sac at L4/5 posteriorly (Figure 3(a)). After removing the ligament (Figure 3(b)), both L5 roots were decompressed perfectly. A hematoma was found inside the ligamentum flavum (Figure 4). After surgery, her symptoms immediately resolved.

## 3. Discussion

Our patient recovered rapidly after surgical removal of the lesion and the previously reported patients also recovered immediately after surgery. To our knowledge, 28 cases of lumbar LFH [1, 2, 4–24] have been reported (Table 1) and all these patients underwent surgical management. However, in the majority of these cases, it was impossible to diagnose LFH prior to surgery [3–5].

The mechanism of development of LFH has not yet been identified [3, 4, 20, 25]. Although minor trauma or laceration of the ligamentum flavum frequently occurs during lumbar punctures for epidural or intrathecal injections, there were some cases of LFH without minor trauma and LFH due to injections that have never been reported [24]. The ligamentum flavum is poorly vascularized, and only a few small vessels pass through it. However, Yayama et al. [26] investigated the histological and immunohistochemical features of degenerative changes in the ligamentum flavum and reported that microangiogenesis was significant around the area of ruptured elastic fibers and collagen fibrils. Tamura et al. [3] reported that degeneration of the ligamentum flavum could potentiate hematoma. In addition, surgical procedures were performed for all cases described in previous reports and nonoperative therapy was not successful—as in our patient. We presume, once a hematoma forms in the ligamentum flavum, it is unable to disperse, due to the elastic fiber and collagen composition of the ligamentum flavum. In addition, histopathological examination after our patient's operation showed there was necrotic ligament tissue around the hematoma, suggesting that there were few blood vessels around the hematoma. That might be the reason why LFH shows differential signal patterns in MRI from usual hematoma including epidural hematoma [2]. In our case, the intensity on T2-weighted imaging of the 2^nd^ MRI was extended than the 1^st^ without changing her symptoms, suggesting that oxyhemoglobin in hematoma got more, not further bleeding. This oxidation procedure is equal to the acute phase of common hematoma, indicating that the speed of hemoglobin oxidation in LFH was very slow.

The biggest challenge faced by clinicians is the difficulty in the diagnosis of this condition, as differentiating LFH from epidural tumours using MR imaging is considered virtually impossible. Keynan et al. [17] reported that an accurate preoperative diagnosis was difficult even on MRI and was only verified by histologic examination after surgical removal. This is because the hematoma may have different signal intensities in relation to the stages of hemoglobin breakdown during the clotting process [2, 6]. Drawing evidence from previous case reports and imaging, three patterns of enhancement of LFH masses have been identified: enhancement of the whole mass [2, 4, 6], enhancement the peripheral section [3, 27], and no enhancement [24, 28]. In our case, the MRI without Gd contrast was carried out in a previous hospital. Therefore, we carried out a second MRI again with Gd contrast two weeks after the first MRI. There were some differences between the first and second time points which appeared to depend on the period of time that had passed since her injury. On imaging, we noted that the mass was not enhanced after intravenous administration of gadolinium (Figure 2(d)).

We propose that differences between the first and second MRI scans can help in diagnosing LFH. However, at the present it is not clear what the appropriate time period between the first and second MRI scans should be. Such an approach may not be possible if the patient had severe symptoms requiring emergency surgery. Nevertheless, this is the first study to highlight the potential importance of differences between the first and second MRI for LFH diagnosis. Carrying out MRI scans in succession may support the diagnostic process for LFH.

## 4. Conclusion

Our patient with lumbar LFH underwent decompression surgery and her symptoms immediately resolved. Following a review of previous cases, we suggest that consecutive MRI scanning may support the diagnostic process for LFH.

## Figures and Tables

**Table 1:** Reported cases of lumbar ligamentum flavum hematoma.

Authors	Patient gender	Age (year)	Level
Sweasey et al. [7]	M	43	L4/5
	M	60	L2/3
Baker and Hanson [8]	F	58	L5/S1
Cruz-Conde et al. [9]	M	57	L4/5
Mahallati et al. [10]	M	30	L3/4
Minamide et al. [4]	M	76	L3/4
Hirakawa et al. [11]	M	50	L4/5
Yuceer et al. [12]	M	67	L2/3
Chi et al. [13]	M	64	L3/4
Mizuno et al. [14]	F	45	L4/5
Yamaguchi et al. [15]	M	62	L4/5
Albanese et al. [16]	F	70	L1/2
Keynan et al. [17]	F	75	L3/4
Shimada et al. [18]	F	83	L2-4
Spuck et al. [19]	F	64	L2/3
	M	62	L4/5
Gazzeri et al. [20]	F	59	L3/4
Kotil and Bilge [21]	M	74	L4/5
	M	80	L4/5
Kono et al. [22]	M	64	L4/5
Miyakoshi et al. [23]	M	71	L3-5
Takahashi et al. [24]	F	53	L3/4
	M	61	L5/S1
Ohba et al. [1]	M	52	L5/S1
Ghent et al. [6]	M	62	L3/4
Liu et al. [2]	M	76	L4/5
Ozdemir et al. [5]	M	63	L2/3
Ishimoto et al. (2017)	F	71	L4/5
